# A bacterial metabolite ameliorates periodontal pathogen-induced gingival epithelial barrier disruption via GPR40 signaling

**DOI:** 10.1038/s41598-018-27408-y

**Published:** 2018-06-13

**Authors:** Miki Yamada, Naoki Takahashi, Yumi Matsuda, Keisuke Sato, Mai Yokoji, Benso Sulijaya, Tomoki Maekawa, Tatsuo Ushiki, Yoshikazu Mikami, Manabu Hayatsu, Yusuke Mizutani, Shigenobu Kishino, Jun Ogawa, Makoto Arita, Koichi Tabeta, Takeyasu Maeda, Kazuhisa Yamazaki

**Affiliations:** 1 Research Unit for Oral-Systemic Connection, Division of Oral Science for Health Promotion, Niigata University Graduate School of Medical and Dental Sciences, Niigata, Japan; 2 Research Center for Advanced Oral Science, Niigata University Graduate School of Medical and Dental Sciences, Niigata, Japan; 3 Division of Microscopic Anatomy and Bio-imaging, Niigata University Graduate School of Medical and Dental Sciences, Niigata, Japan; 4 Division of Applied Life Sciences, Graduate School of Agriculture, Kyoto University, Kyoto, Japan; 5 Laboratory for Metabolomics, RIKEN Center for Integrative Medical Sciences, Kanagawa, Japan; 6 Division of Periodontology, Department of Oral Biological Science, Niigata University Faculty of Dentistry, Niigata, Japan

## Abstract

Several studies have demonstrated the remarkable properties of microbiota and their metabolites in the pathogenesis of several inflammatory diseases. 10-Hydroxy-*cis*-12-octadecenoic acid (HYA), a bioactive metabolite generated by probiotic microorganisms during the process of fatty acid metabolism, has been studied for its protective effects against epithelial barrier impairment in the intestines. Herein, we examined the effect of HYA on gingival epithelial barrier function and its possible application for the prevention and treatment of periodontal disease. We found that GPR40, a fatty acid receptor, was expressed on gingival epithelial cells; activation of GPR40 by HYA significantly inhibited barrier impairment induced by *Porphyromonas gingivalis*, a representative periodontopathic bacterium. The degradation of E-cadherin and beta-catenin, basic components of the epithelial barrier, was prevented in a GPR40-dependent manner *in vitro*. Oral inoculation of HYA in a mouse experimental periodontitis model suppressed the bacteria-induced degradation of E-cadherin and subsequent inflammatory cytokine production in the gingival tissue. Collectively, these results suggest that HYA exerts a protective function, through GPR40 signaling, against periodontopathic bacteria-induced gingival epithelial barrier impairment and contributes to the suppression of inflammatory responses in periodontal diseases.

## Introduction

Increasing evidence has indicated that the microbiota and its metabolites are key orchestrators of host pathophysiology through the modulation of metabolism, inflammation, and immune responses^1–3^. We have recently reported that several *Lactobacillus* strains have the ability to convert linoleic acid to oleic acid via intermediate metabolites, such as 10-hydroxy-*cis*-12-octadecenoic acid (HYA), 10-hydroxyoctadecanoic acid (HYB), 10-oxo-*cis*-12-octadecenoic acid (KetoA), 10-oxo-octadecanoic acid (KetoB), and 10-oxo-*trans*-11-octadecenoic acids (KetoC); have beneficial effects on the regulation of host energy metabolism and immunomodulatory activities^4–6^. Furthermore, Miyamoto *et al*. reported that *Lactobacillus*-derived HYA, but not HYB, plays a protective role against intestinal injury and inflammation by ameliorating intestinal barrier impairment via activation of G protein-coupled receptor 40 (GPR40)^7^. GPR40, a receptor for a range of medium- to long-chain saturated and unsaturated fatty acids, was originally reported to be highly expressed in pancreatic beta cells and regulates insulin secretion and blood glucose^8^. Subsequent studies have indicated that GPR40 is also expressed in different cell types, including epithelial cells, and is involved in the pathogenesis of several diseases such as inflammatory bowel disease, asthma, and kidney disease^7,9,10^. However, there are no reports regarding the expression of GPR40 in gingival epithelial cells and its function in inflammatory oral disorders.

Periodontal disease, or periodontitis, is defined as an infectious and inflammatory disease affecting the supporting tissues of the teeth. More than 700 bacterial species have been identified in the oral cavity, some of which are known as periodontopathic bacteria^11,12^. *Porphyromonas gingivalis* (*P. gingivalis*) has been implicated as a major etiological pathogen in periodontitis, because of a variety of virulence factors, including fimbriae^13,14^, lipopolysaccharides^15,16^, capsule^17,18^, and proteases^19,20^. Gingipains are cysteine proteases secreted by *P. gingivalis* and are considered as the major causative agent in periodontal disease, leading to destruction of the supporting tissues of the periodontium by degradation and cleavage of the extracellular matrix and cell surface proteins^21^. Katz *et al*. have reported that *P. gingivalis* and its purified gingipains breakdown the epithelial barrier proteins which form, in part, the adherens and tight junctions^22,23^. Disruption of the gingival epithelial barrier by specific proteases and the subsequent penetration of exogenous pathogens into the host tissues facilitate the progression of periodontal breakdown^24^. Hence, gingival epithelial cells play crucial roles in the initiation and progression of periodontal diseases by acting as a physical barrier between the outside and the host.

E-cadherin, a transmembrane glycoprotein expressed by epithelial cells, is an essential adhesion molecule for barrier formation by establishing strong cell-cell contacts between epithelial cells^25^. Beta-catenin is a complex partner of E-cadherin and plays a key role in the maintenance of adherence junction complexes by linking the cytoskeleton with E-cadherin^26^. Disruption of the functions of the barrier, which is composed of an E-cadherin/beta-catenin complex, is associated with a variety of human diseases^27^. Recent studies have demonstrated a decrease in E-cadherin expression levels in the gingival epithelium in periodontitis, suggesting the clinical importance of E-cadherin in the pathogenesis of periodontal diseases^28,29^.

Herein, we explored the impact of bioactive metabolites on gingival epithelial barrier impairment induced by periodontopathic bacteria. This study demonstrates that epithelial barrier impairment is ameliorated by HYA, partially via effects on GPR40 signaling that subsequently inhibits the degradation of adhesion proteins. Furthermore, oral administration of HYA in a mouse model of experimental periodontitis inhibited the degradation of E-cadherin and subsequent inflammatory responses, thereby indicating the protective role of HYA in periodontal disease.

## Results

### Expression of GPR40 by gingival epithelial cells

**Figure 1:**
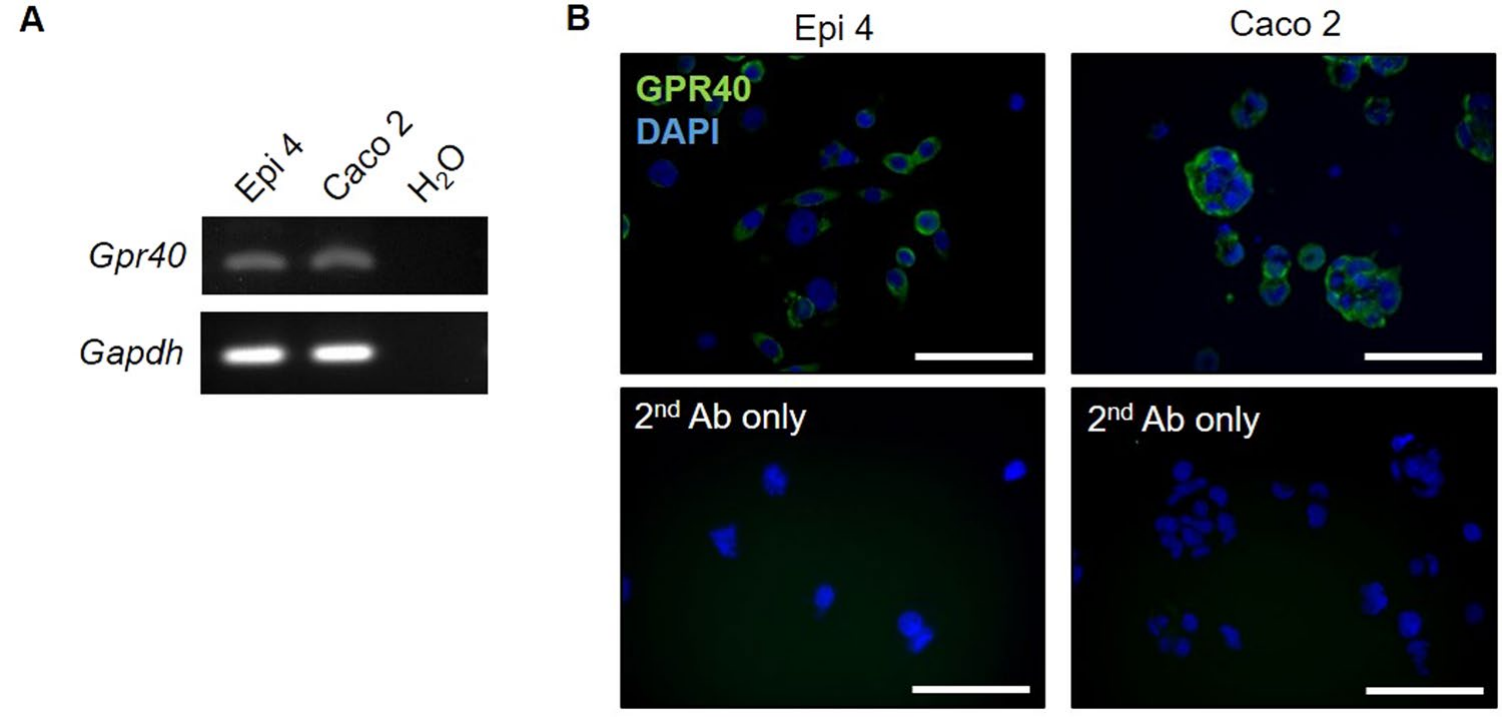
Expression of GPR40 in gingival epithelial cells. (**A**) Validation of *Gpr40* mRNA expression by RT-PCR. *Gapdh* was used as an internal control. Full-length gels are presented in Supplementary Fig. 1. (**B**) Representative immunofluorescence staining of epithelial GPR40. Nuclei were counterstained using DAPI. The lower panels represent sections without primary antibody that served as negative controls. Scale bars: 100 μm.

First, we validated GPR40 gene expression in a gingival epithelial cell line (Epi 4) and an intestinal epithelial cell line (Caco 2; positive control) by semi-quantitative reverse transcription polymerase chain reaction (RT-PCR) (Fig. 1A). Next, protein expression of GPR40 in Epi 4 cells was confirmed by immunofluorescence staining (Fig. 1B). Our findings suggest that GPR40 is expressed in the gingival epithelial cells as both mRNA and protein.

### Protective effect of HYA-induced GPR40 signaling on epithelial barrier impairment

**Figure 2:**
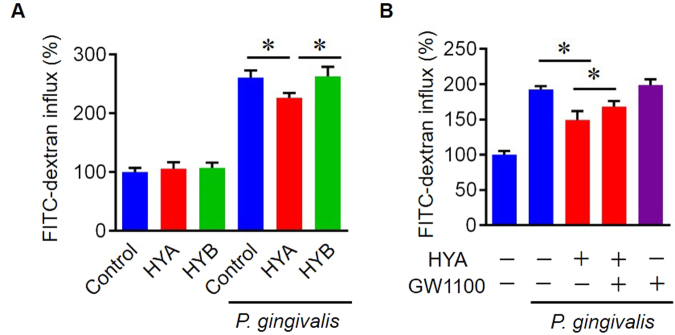
HYA treatment suppresses *P. gingivalis*-induced barrier dysfunction via GPR40. (**A**) Epi 4 cells seeded in the upper compartment were pretreated with the indicated metabolites (5 μM) for 30 min, followed by stimulation with live *P. gingivalis* (MOI: 100) for 4 h. The paracellular permeability of Epi 4 cells was measured by fluorescence after adding FITC-dextran to the upper compartment for 2 h. (**B**) GW1100 (5 μM) was added to the indicated samples prior to HYA/*P. gingivalis* treatment (n = 4 in each group). All data are presented as mean ± SD (*p < 0.05 as indicated).

To examine a possible role of bioactive metabolites in gingival epithelial barrier function, we performed an *in vitro* permeability assay using fluorescein isothiocyanate (FITC) -conjugated dextran after the optimization of HYA concentration (Supplementary Fig. 2). The epithelial barrier impairment induced by *P. gingivalis* was significantly inhibited by pretreatment with HYA but not HYB (Fig. 2A). Pretreatment with the selective GPR40 antagonist GW1100 partially diminished the protective property of HYA, indicating a GPR40-dependent mechanism (Fig. 2B). Taken together, these results demonstrate that HYA prevents *P. gingivalis*-induced barrier impairment, via its activation of GPR40 signaling.

### Transmission electron microscopy (TEM) images of the junctional complex of the epithelium

**Figure 3:**
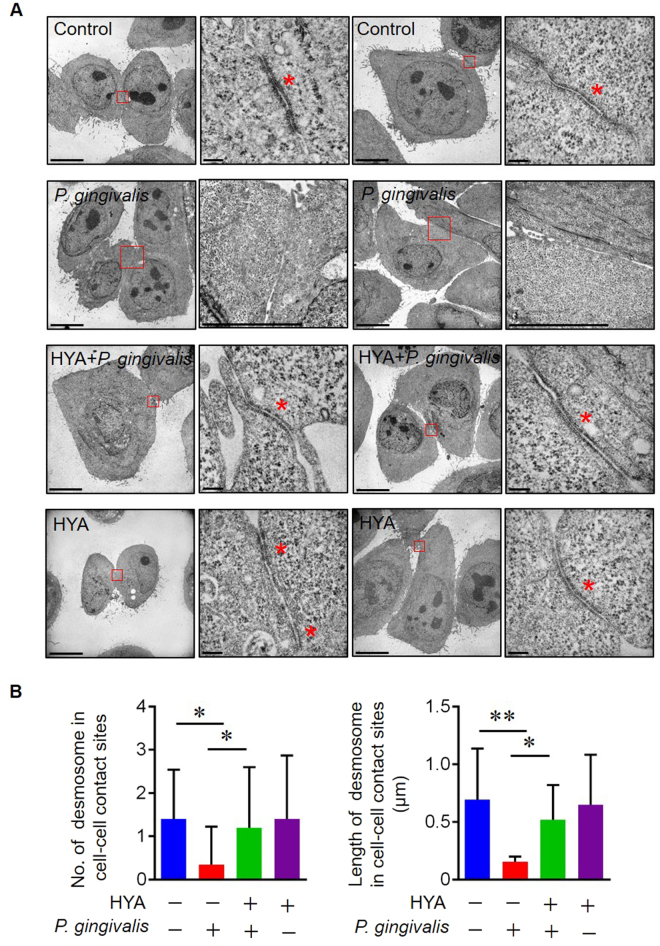
TEM imaging of the junctional complex of Epi 4 cells. (**A**) TEM images of Epi 4 cells after the indicated treatment (2 sets of representative images from each group. Left panels; low magnification, scale bars: 10 µm, right panels; high magnification, scale bars: 200 nm) Asterisks indicate epithelial junctions of the Epi 4 cells. (**B**) Measurements of number of desmosome in cell-cell contact sites (left) and length of desmosome in cell-cell contact sites (n = 20 in each group). All data are presented as mean ± SD. *p < 0.05, **p < 0.01 as indicated, by ANOVA.

As morphological alterations of cell-cell junctional complexes of epithelial cells affect the integrity and barrier function of the epithelium, we performed ultrastructural observations of cultured Epi 4 cells by TEM. Interestingly, cell-cell adhesion structures were dramatically disrupted by incubation with *P. gingivalis*, and the disruption was clearly diminished by pretreatment with HYA (Fig. 3A,B). No morphological changes were observed for Epi 4 cells treated with HYA only. These results imply that HYA inhibits the degradation of adhesion structures induced by *P. gingivalis*.

### Inhibitory effect of HYA on the degradation of adhesion molecules

**Figure 4:**
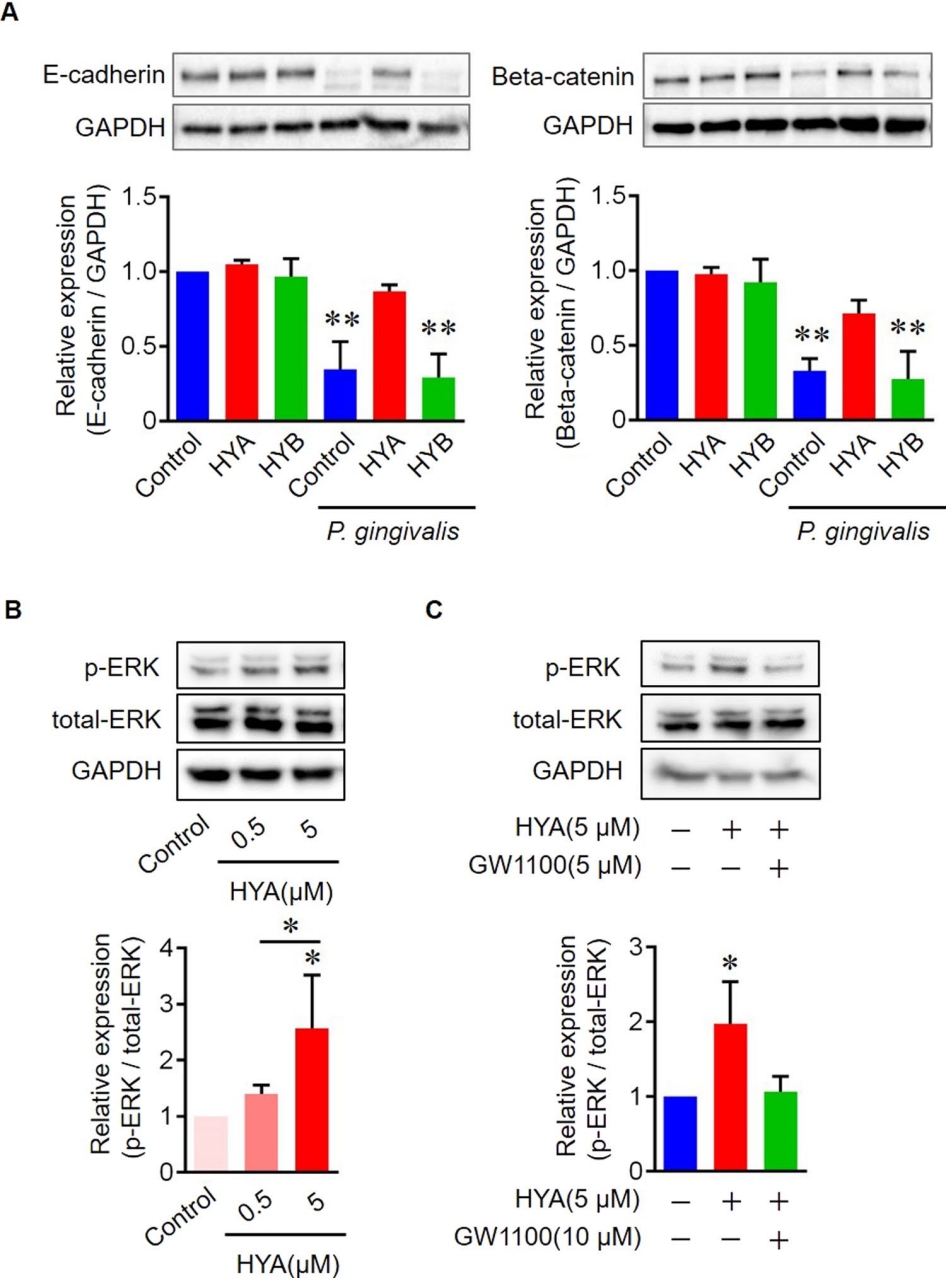
Inhibitory effect of HYA on the degradation of adhesion molecules *in vitro*. (**A**) Western blots and quantification of adherence junction proteins in Epi 4 cells. Epi 4 cells were stimulated with or without *P. gingivalis* (MOI: 100) for 4 h, with or without HYA/HYB preincubation (5 µM) for 30 min. The band signal of each target protein was normalized to GAPDH. Full-length blots are presented in Supplementary Fig. 5. (**B**) Western blots and quantification of p-ERK. Epi 4 cells were stimulated with HYA (0.5, 5 µM) for 30 min. The band signal of p-ERK was normalized to total-ERK. Full-length blots are presented in Supplementary Fig. 6. (**C**) Western blots and quantification of p-ERK. Epi 4 cells were stimulated with HYA (5 µM) for 30 min, with or without GW1100 preincubation (5 µM) for 30 min. The band signal of p-ERK was normalized to total-ERK. Full-length blots are presented in Supplementary Fig. 7 (n = 3 in each group). All data are presented as mean ± SD. *p < 0.05, **p < 0.01 as indicated, by ANOVA.

To elucidate the molecular mechanisms underlying the inhibitory effect of HYA against damage to the adherence junction, we focused on the E*-*cadherin/beta*-*catenin complex, which plays an important role as a major component of the adherence junction. Western blot analysis showed that *P. gingivalis* induced the degradation of both E-cadherin and beta-catenin proteins, and pretreatment with HYA blocked this degradation substantially (Fig. 4A). No alteration on *E*-*cadherin/Beta*-*catenin* mRNA level was observed by real-time PCR in all groups (Supplementary Fig. 8), suggesting that HYA promotes the proteolytic resistance of E-cadherin/Beta-catenin against *P. gingivalis* by inducing post-translational modifications. Extracellular signal-regulated kinase (ERK) is a key mediator in post-translational modifications^30^, particularly in barrier function-related proteins^31–33^. We demonstrated that HYA phosphorylated ERK in a dose-dependent manner, and pretreatment with GW1100 significantly inhibits the phosphorylation (Fig. 4B,C), suggesting that HYA prevents the degradation of E*-*cadherin/beta*-*catenin proteins in Epi 4 cells by post-translational mechanisms via HYA-GPR40-pERK intracellular pathways.

### HYA reduces local inflammatory cytokine production in gingival tissue *in vivo*

**Figure 5:**
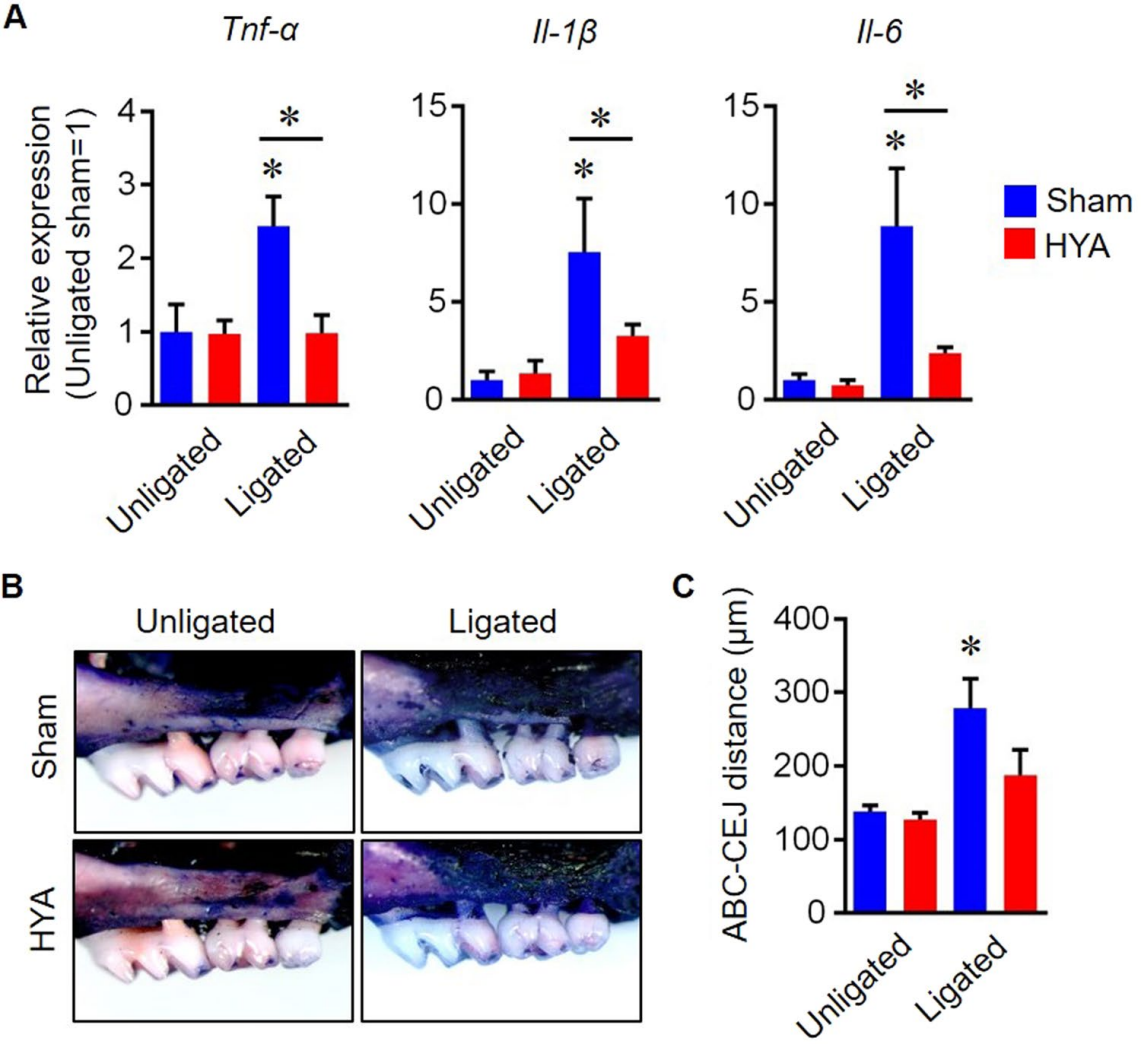
HYA reduces local inflammatory cytokine production in gingival tissue. (**A**) Quantification of inflammatory cytokine mRNA expression in gingival tissue. (**B**) Representative stereoscope images of defleshed maxilla from each group on day 14. (**C**) Quantification of alveolar bone loss measured by the distance from CEJ to ABC (n = 6 in each group). All data are presented as mean ± SD. p < 0.01 versus unligated wild-type or as indicated, by ANOVA.

**Figure 6:**
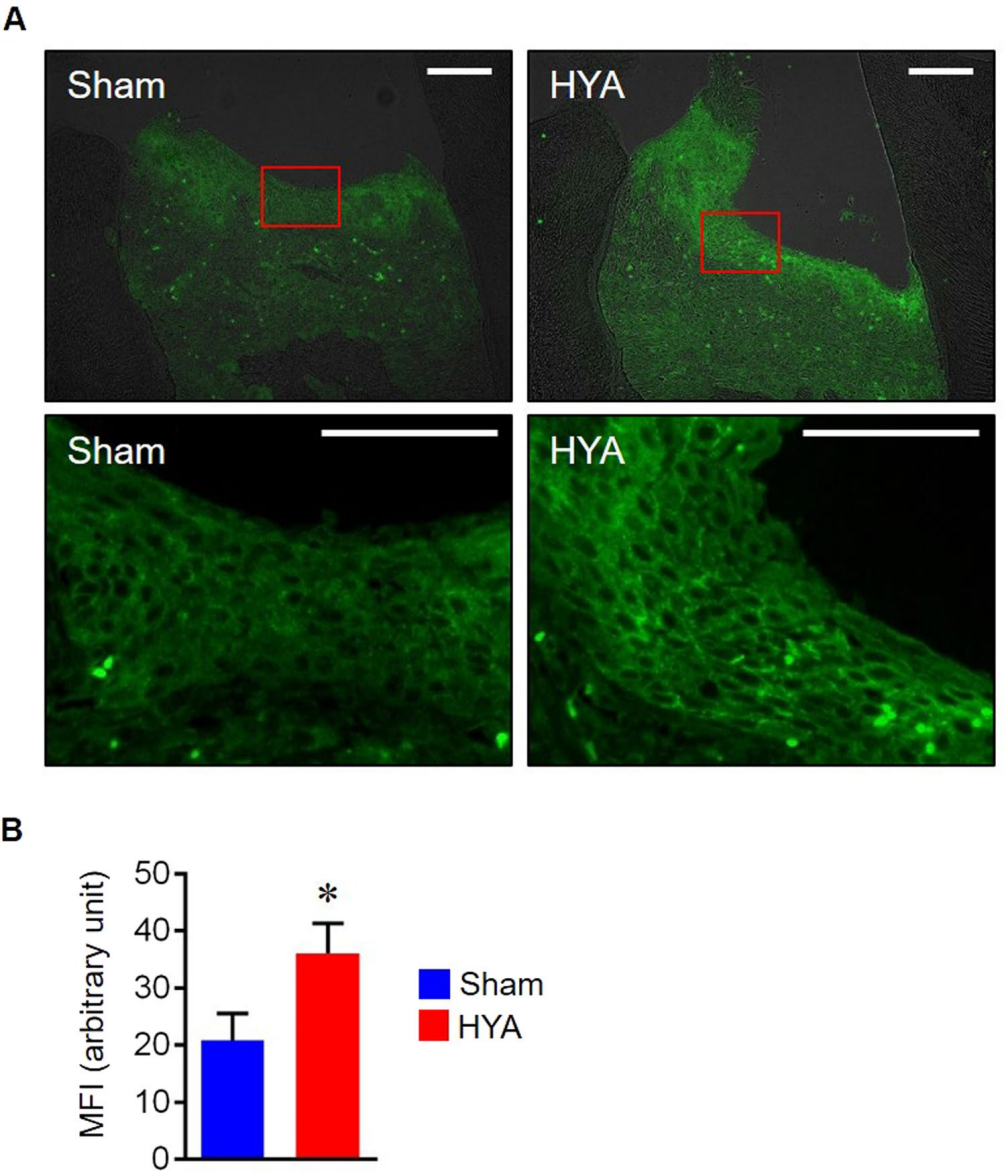
HYA suppresses the degradation of gingival E-cadherin in mice. (**A**) Representative immunofluorescence staining for E-cadherin in gingival tissues of ligated mice. Lower panels represent magnified views of the boxed areas. Scale bars: 100 μm. (**B**) Mean fluorescence intensity (MFI) of E-cadherin in gingival epithelial layer (n = 3 in each group). All data are presented as mean ± SD. *p < 0.05, versus sham group, by ANOVA.

In order to examine the clinical relevance of HYA in periodontitis, an *in vivo* study was carried out using a mouse experimental periodontal disease model. After validation of epithelial GPR40 expression in the gingiva, periodontitis was induced by applying a ligature on the molars with repeated oral inoculation of *P. gingivalis* **(**Supplementary Figs 9,10A). The mRNA expression levels of inflammatory cytokines such as *IL-1β*, *TNF-α* and *IL-6* in gingival tissues of the ligated HYA-treated group were significantly decreased in comparison with those of the ligated sham-treated group, with a tendency of suppression of alveolar bone destruction (Fig. 5A–C). The ligated HYA-treated group showed a higher immunofluorescence intensity for E-cadherin in the epithelium when compared to that of the ligated sham-treated group (Fig. 6A,B**)**. No differences of E-cadherin intensity were observed between the unligated groups **(**Supplementary Fig. 10B). In consistent with *in vitro* study, no alteration of *E-cadherin* mRNA expression was observed for all groups (Supplementary Fig. 10D). Collectively, these results indicate that HYA reinforces gingival epithelial barrier function by inhibiting E-cadherin degradation, resulting in the suppression of periodontal inflammatory responses and subsequent alveolar bone destruction *in vivo*.

## Discussion

The barrier function of the epithelium is important in host defense against invading pathogens. In this study, we demonstrated that the gingival epithelial cells express a fatty acid receptor, GPR40; activation of this receptor by HYA prevents the impairment of the epithelial barrier that is caused by periodontopathic bacteria. Furthermore, the findings of our *in vivo* studies suggest that treatment with HYA has a beneficial role in the prevention of initiation/progression of periodontal diseases.

It is becoming increasingly clear that metabolites, intermediates of metabolism, are linked to human health and disease^2,3^. In the gut, increased intestinal permeability of the epithelial barrier, also known as “leaky gut” is associated with several gastrointestinal and systemic disorders^34–36^. A variety of microbiota- and diet-derived metabolites such as indole^37,38^, acetate^39^, and butyrate^40^ are noted to have beneficial effects against intestinal epithelial barrier dysfunction. Similarly, the physiological functions of metabolites in the oral cavity have been documented in several studies. Recent human metabolomics research has revealed significant correlations between the levels of several metabolites in saliva and the severity of periodontal disease, suggesting a possible involvement of metabolites in periodontitis^41,42^. Further investigation will provide mechanistic insights into how these bioactive metabolites contribute to the pathophysiology of periodontal diseases.

To explore the mechanisms underlying the protective effects of HYA against *P. gingivalis*-induced epithelial barrier impairment, we first evaluated the direct effects of HYA on *P. gingivalis* and its proteolytic activity against epithelial barrier-related proteins. Fatty acids have anti-proteolytic activity and antimicrobial activity against several oral pathogens^43,44^; nevertheless, we failed to demonstrate these properties of HYA in the present study (Supplementary Figs 3, 4 and 10E). Therefore, we focused on the epithelial GPR40 receptor-dependent regulation of barrier function, and we found that the breakdown of barrier function was inhibited partially in a GPR40-dependent manner (Fig. 2B). It is well documented that the activation of epithelial GPR signaling is crucial for a wide variety of physiological and pathological processes in several diseases. It has been reported that the intestinal epithelial barrier is modulated by regulation of TNFR2 expression via the GPR40-MEK-ERK pathway^7^. Gras *et al*. have demonstrated a proliferative effect on human bronchial epithelial cells via GPR40 receptor activation, involving an intracellular calcium-signaling pathway^10^. The activation of GPR40 in renal epithelial cells attenuates apoptosis by inhibiting the activation of the Sic/EGFR/ERK signaling pathway and the nuclear activation of NF-κB^9^. Elucidating the signaling pathways involved in GPR40 activation could help to unveil the underlying mechanisms responsible for the observations made during the present study.

In this study, we demonstrated that HYA influences epithelial barrier function at the protein level, but not at the transcriptional level (Fig. 4A, Supplementary Fig. 8). Although Miyamoto *et al*. showed that HYA modulates the expression levels of barrier function-related genes in epithelial colorectal adenocarcinoma cells (Caco 2), we did not observe any transcriptional regulation in Epi 4 cells. This difference may be explained by differences in the types of epithelial cells (oral vs intestinal) and/or the stimulants (inflammatory cytokine vs microorganisms) used in the studies. In fact, previous studies have reported differences in biological characterization between gingival and intestinal epithelial cells^45^, implying that HYA regulates gingival epithelial barrier by different molecular mechanisms.

Regarding the partial effect of the GPR40 inhibitor on the epithelial permeability assay (Fig. 2), it may be because fatty acids including HYA are not recognized only by GPR40^46^. Given the expressions of other G-couple protein such as GPR41, GPR43 and GPR120 in epithelial cells^7^, these receptors might be also activated by HYA; therefore, the specific inhibition against GPR40 by GW1100 showed only partial effect.

Post-translational modifications of proteins (e.g., phosphorylation, glycosylation, and acetylation) dramatically influence protein stability, structure, and localization^47,48^. Multiple post-translational modifications of E-cadherin have been extensively studied regarding its stability and maturation. McEwen *et al*. reported that phosphorylation of the beta-catenin-binding domain of E-cadherin is responsible for intercellular adhesion by stabilizing the cadherin at the cell surface^49^. Glycosylation of E-cadherin directly influences the maturity of the adherence junction by affecting its molecular organization^50,51^. The glycosylated proteins exhibit higher resistance to proteolytic degradation than that of their original forms^52–54^, suggesting that post-translational modifications of E-cadherin affect its proteolytic sensitivity. ERK is one of the three major subfamilies of the mitogen-activated protein kinase (MAPK) signaling pathways, and plays an important role in multiple post-translational modifications on barrier function-related proteins^31–33^. Our findings in this study indicate that HYA facilitates the post-translational modifications on E-cadherin in a GPR40-dependant manner via ERK activation. Taken together, these findings suggest that epithelial HYA-GPR40-ERK signaling may induce the post-transcriptional modulation of E-cadherin, resulting in more resistance of E-cadherin to *P. gingivalis* proteolytic activity. Our proposed mechanism was illustrated in Supplementary Fig. 11. Both downstream targets of ERK phosphorylation and distinct mechanisms of post translational modification of E-cadherin remain to be resolved.

In summary, this study demonstrates for the first time the presence of GPR40 in gingival epithelial cells and its beneficial effects against epithelial barrier impairment. Furthermore, this *in vivo* study also indicates that HYA is capable of ameliorating gingival epithelial barrier disruption and preventing the inflammatory responses of periodontal tissue. A new therapeutic approach for periodontitis, which enhances epithelial barrier function, might offer advantages over conventional periodontal treatment (e.g., mechanical plaque control and root planing). In addition, reducing antibiotic use in periodontal therapy by manipulating host defense using HYA may potentially lead to minimization of the risk of antibiotic resistance in the coming super-aged society.

## Methods

### Reagents and antibodies

10-hydroxy-*cis*-12-octadecenoic acid (HYA) and 10-hydroxyoctadecanoic acid (HYB) were synthesized according to methods published previously^4^. Anti-GPR40 antibody was purchased from GeneTex Inc. (San Antonio, TX, USA). Anti-E-cadherin, anti-Beta-catenin, anti-ERK and anti-phosphorylated ERK antibodies were obtained from Cell Signaling Technology (Danvers, MA, USA). Rabbit anti-mouse glyceraldehyde 3-phosphate dehydrogenase (GAPDH; Cell Signaling Technology) and peroxidase-labeled anti-rabbit IgG antibody (Cell Signaling Technology) were used for Western blotting experiments. A selective GPR40 antagonist, GW1100, was purchased from Cayman Chemical (Ann Arbor, MI, USA).

### Cell preparation and culture

The Epi 4 simian virus 40 immortalized human gingival epithelial cell line was kindly provided by Prof. Murakami (Osaka University, Japan) and maintained as previously described^15,55^. The Caco 2 human intestinal epithelial cell line was obtained from the Riken BioResource Center (Tsukuba, Japan) and cultured in Dulbecco’s modified Eagle’s medium supplemented with 10% fetal bovine serum, 100 U/mL penicillin, and 100 µg/mL streptomycin.

### Bacterial strains and culture conditions

*P. gingivalis* strain W83 was cultured in modified Gifu anaerobic medium (GAM) broth (Nissui, Tokyo, Japan) in an anaerobic jar (Becton Dickinson Microbiology System, Cockeysville, MD, USA), in the presence of an AnaeroPackTM (Mitsubishi Gas Chemical Co. Inc., Tokyo, Japan) at 37 °C for 48 h. Bacterial suspensions were prepared in phosphate-buffered saline (PBS) without Mg^2+^/Ca^2+^, using established growth curves and spectrophotometric analysis. The number of colony-forming units (CFU) was standardized by measuring optical density (OD) at 600 nm.

### Reverse transcription polymerase chain reaction (RT-PCR) and gel electrophoresis

Total RNA was isolated from cells and gingival tissues using TRI Reagent® (Molecular Research Center, Inc., Cincinnati, OH, USA). cDNA was synthesized using a Transcriptor Universal cDNA Master (Roche Molecular Systems, Inc., Branchburg, NJ, USA). Semi-quantitative RT-PCR was performed in a 20 μL reaction volume with GoTaq polymerase (Promega Corporation, Madison, WI, USA) using the following protocol: predenaturation at 94 °C for 5 min followed by 30 cycles of denaturation at 94 °C for 15 s, annealing at 60 °C for 15 s, extension at 72 °C for 30 s, and a final extension step at 72 °C for 10 min using a GeneAmp® PCR System 7700 (Applied Biosystems, Carlsbad, CA, USA). The PCR products were run on 1.5% agarose gels and visualized using SYBR® Safe DNA (Invitrogen Corporation, Carlsbad, CA, USA).

### Quantitative PCR

Quantitative PCR was performed on a LightCycler*®* 480 (Roche Molecular Systems) using a FastStart Essential DNA Green Master (Roche Molecular Systems). The relative expression levels of each mRNA were normalized to that of *Gapdh* mRNA using the *delta delta* Ct method^56^. The custom-designed oligonucleotide sequences (Thermo Fisher Scientific, MA, USA) used for both semi-quantitative RT-PCR and quantitative PCR are summarized in Tables 1 and 2, respectively.

**Table 1:** Primer sequences for *in vitro* experiments.

Gene	Forward	Reverse
*Gapdh*	ACCAAATCCGTTGACTCCGAC	TTCGACAGTCAGCCGCATCT
*Gpr40*	AGTGTGGTGCTTAATCCGCT	AGTGGCGTTACTTCTGGGAC
*E-cadherin*	CTTGGAGCCGCAGCCTCT	ACACCATCTGTGCCCACTTT
*Beta-catenin*	ACGGAGGAAGGTCTGAGGAG	GCCGCTTTTCTGTCTGGTTC

**Table 2:** Primer sequences for *in vivo* experiments.

Gene	Forward	Reverse
*Gapdh*	TCAACAGCAACTCCCACTCTT	ACCCTGTTGCTGTAGCCGTAT
*Tnf-alpha*	GATCGGTCCCCAAAGGGATG	TTGACGGCAGAGAGGAGGTT
*Il-1beta*	TGCCACCTTTTGACAGTGATG	AAGGTCCACGGGAAAGACAC
*Il-6*	CCGGAGAGGAGACTTCACAG	TCTGAAGGACTCTGGCTTTGT
*Gpr40*	CACTTTGCTCCCCTCTACGC	GATGGCTTGGTACCCGAAGG
*E-cadherin*	CTACAGCATCACCGGCCAA	CCACCGCTTCCCCATTTGA

### Immunostaining

The Epi 4 and Caco 2 cells were seeded in a Lab-Tek™ Chamber Slide (Nunc, Rochester, NY, USA) at a density of 5 × 10^4^ cells/well for immunofluorescence staining. The attached cells were fixed with 4% paraformaldehyde, washed in Tris-buffered saline, and stained with anti-GPR40 (1: 200) and Alexa Fluor 488-conjugated anti-rabbit secondary antibodies (1: 200) (Abcam, Cambridge, UK). The slides were mounted using VECTASHIELD® HardSet™ Mounting Medium with DAPI (Vector Laboratories, Burlingame, CA, USA) and analyzed by fluorescence microscopy (Biozero BZ-8000; Keyence Corporation, Osaka, Japan).

For the immunostaining of periodontal tissues, samples were fixed, decalcified, embedded, and sectioned as described previously^57^. Tissue sections were deparaffinized and incubated with anti-GPR40 (1: 200) or anti-E-cadherin antibody (1: 200) at 4 °C overnight. Immunoreactivity of GPR40 was detected with biotinylated chicken anti-rabbit immunoglobulin (1: 200) (Abcam) in an avidin-biotin-immunoperoxidase system (Vector Laboratories). E-cadherin was visualized using Alexa Fluor 488-conjugated anti-rabbit secondary antibody (1: 200) (Abcam). The specificity of E-cadherin antibody for gingival tissues was validated as in Supplementary Fig. 10 C. The quantification of the immunofluorescent staining was performed using ImageJ software (National Institute of Health, Bethesda, MD, USA). Briefly, the mean fluorescence intensity corresponding to E-cadherin (green color) in gingival epithelial layers was compared between groups.

### Epithelial barrier function assay

An *in vitro* epithelial permeability assay to assess barrier function was performed with FITC-conjugated dextran using Millicell® 24-well Hanging Cell Culture Inserts (EMD Millipore Corporation, Billerica, MA, USA) as reported previously^58,59^. Epi 4 cells were cultured in the upper compartments at a concentration of 5 × 10^4^ cells/well; 5 μl of 10 mg/ml FITC-dextran (average molecular weight, 3,000 to 5,000; Sigma-Aldrich) was added to the upper compartments of the inserts. The medium was collected from the lower chamber compartments 2 h after FITC-dextran addition, and fluorescence intensity was measured using an EMax Plus plate reader (Molecular Devices, Sunnyvale, CA, USA) at 485 nm excitation and 520 nm emission wavelength.

### TEM imaging

The samples were fixed with 1.0% glutaraldehyde in Dulbecco’s PBS (Thermo Fisher Scientific) at 4 °C for 20 min; they were subsequently post-fixed with 1% osmium tetroxide for 20 min at 4 °C. The fixed samples were washed with distilled water for 10 min, three times, and stained with uranyl acetate for 2 h at 4 °C. The stained samples were washed with distilled water for 10 min, three times, dehydrated in a graded ethanol series, and embedded in Epon 812 (Nisshin EM Co. Ltd., Tokyo, Japan). Ultrathin sections (70 nm) were cut on an ultramicrotome (Ultracut-N, Reichert-Jung, Vienna, Austria) and placed on 150-mesh copper grids. The sections were stained with uranyl acetate (for 10 min) and lead citrate (for 5 min), and observed using a transmission electron microscope (H-7650, Hitachi, Tokyo, Japan) at an accelerating voltage of 80 kV. The quantification of TEM images was performed by referring to previous publications^60,61^. Briefly, 20 randomly selected cell-cell contact sites were captured, and the number of desmosome-like structures and their length in each contact sites were measured in a blind manner.

### Western blotting

Total protein was extracted using M-PER Mammalian Protein Extraction Reagent (Thermo Fisher Scientific) with Halt Protease Inhibitor Cocktail and Halt Phosphatase Inhibitor Cocktail (Pierce Biotechnology, Rockford, IL, USA). Protein concentration was determined using a Pierce Bicinchoninic Acid Protein Assay Kit (Pierce Biotechnology). Each sample was solubilized in sodium dodecyl sulfate (SDS) sample buffer, separated by SDS-polyacrylamide gel electrophoresis, and transferred to polyvinylidene fluoride membranes (EMD Millipore Corporation). After incubation with the appropriate primary (E-cadherin; 1: 500, Beta-catenin; 1: 500, ERK; 1: 500, p-ERK; 1: 500, GAPDH; 1: 5000) and secondary antibodies (peroxidase-labeled anti-rabbit IgG antibody; 1: 5000), target proteins were detected using ECL Plus Western blotting detection reagents (GE Healthcare) and a LumiVision PRO 400EX system (Aisin Seiki Co., Ltd., Aichi, Japan). The intensity of the signal was quantified using ImageJ software. The intensity of each molecule was expressed after normalization to the GAPDH or total-ERK intensity.

### Mice

All experiments were performed in accordance with the Regulations and Guidelines on Scientific and Ethical Care and Use of Laboratory Animals of the Science Council of Japan, enforced on June 1, 2006, and approved by the Institutional Animal Care and Use Committee at Niigata University (permit number 151-3). Eight-week-old male C57BL/6 mice were purchased from Japan SLC, Inc. (Shizuoka, Japan). All mice were acclimatized under specific pathogen-free conditions and fed regular chow and sterile water throughout the experiment.

### Induction of periodontitis in mice and administration of HYA

Murine experimental periodontitis was induced as described previously with minor modifications^62^. In brief, a 5–0 silk ligature was tied around the maxillary second molar under anesthesia without damaging the surrounding gingiva. During the ligation period, *P. gingivalis* (10^9^ CFU) suspended in 100 µl of 2% carboxymethyl cellulose (Sigma-Aldrich) was given to the mice using a feeding needle every 2 days. The unligated group mice were sham-infected without *P. gingivalis* and served as controls. HYA was administrated via drinking water at a final concentration of 50 mM for 14 days. On day 7, half of the mice with *P. gingivalis* infection underwent ligation; all mice were sacrificed for analysis on day 14. The experimental design of this study is illustrated in Supplementary Fig. 10A.

### Measurement of alveolar bone loss

After defleshing, the bones were subjected to brushing and bleaching. The maxillae were stained with 1% methylene blue to delineate the CEJ and ABC. The distances of the mesial roots of the maxillary second molar from the CEJ to ABC were measured on images obtained with a stereomicroscope (DP2-BSW; OLYMPUS, Tokyo, Japan). Alveolar bone loss measurements were performed in a blind manner.

### Measuring cell viability/cytotoxicity

The Epi 4 cells were seeded into a 96-well plate (5 × 103 cells/well) and incubated in the absence or presence of the indicated concentrations of HYA or HYB. The MTT assay was performed according to the manufacturer’s instructions (Sigma-Aldrich).

### Assessment of antimicrobial activity

*P. gingivalis* bacteria were cultured in GAM broth under anaerobic conditions in the absence or presence of various concentrations of HYA or HYB at 37 °C. OD values (600 nm) were measured at the indicated time points using an EMax Plus plate reader.

### *In vitro* degradation of adhesion proteins

Recombinant human E-cadherin protein (R&D Systems, Inc., MN, USA) was incubated with live *P. gingivalis* with or without the preincubation of metabolites at the indicated concentrations. Electrophoresis was carried out using a Mini-PROTEAN Tetra System (Bio-Rad); 10% SDS-polyacrylamide gels were stained with Coomassie Blue, and the protein bands on gels were detected using an Imaging Scanner. In order to examine the anti-proteolytic properties of metabolites under physiological conditions, a purified E-cadherin protein obtained from the Epi 4 cells was used. Purification was performed by an immunoprecipitation-based method according to the manufacturer’s instructions (Santa Cruz Biotechnology, Dallas, TX, USA). The purified E-cadherin was incubated with live *P. gingivalis* with or without preincubation with various concentrations of HYA or HYB, and then detected by Western blotting using a specific antibody.

### Determination of bacterial accumulation

A sterile paper point (Zipperer Absorbent Paper Points, VDW GmbH, Munich, Germany) was held against the gum line in the oral cavity for 5 s. Bacterial DNA was extracted from these samples using a QIAampDNA Blood Mini Kit (Qiagen, Hilden, Germany). Quantitative real-time PCR was performed with 5 μL of sample DNA in a final volume of 20 μL per reaction using a Fast Start Essential DNA Green Master (Roche) on a LightCycler® 96 System (Roche). The universal 16 S rRNA sequence was amplified by predenaturation at 95 °C for 30 s, followed by 40 cycles at 95 °C for 10 s and at 60 °C for 30 s using a specific primer for universal 16 S rRNA (forward primer 5′-ACTCCTACGGGAGGCAGCAGT-3′; reverse primer 5′-ATTACCGCGGCTGCTGGC-3′). The Ct values obtained from the PCR were converted to gene copy numbers to estimate the amount of bacterial genomes.

### Statistical analysis

All experiments were independently repeated at least twice, on separate days. All data are expressed as the mean ± standard deviation (SD). Statistical analyses were performed using GraphPad Prism (GraphPad Software, Inc., San Diego, CA, USA), and a p-value < 0.05 was considered as statistically significant.

### Data availability statement

The data that support the findings of this study are available from the corresponding author, N.T. and K.Y, upon reasonable request.

## Electronic supplementary material

Supplementary Figures
