# Supplementary material for: A bacterial metabolite ameliorates periodontal pathogen-induced gingival epithelial barrier disruption via GPR40 signaling
Source: Sci Rep. 2018 Jun 13;8:9008. doi: 10.1038/s41598-018-27408-y (PMC5998053; doi:10.1038/s41598-018-27408-y)
Supplement: Supplementary file 1 — Supplementary Figures [file 41598_2018_27408_MOESM1_ESM.pdf]

Supplemental Figures

A bacterial metabolite ameliorates periodontal pathogens-induced gingival epithelial barrier disruption via GPR40 signaling

Miki Yamada[1], Naoki Takahashi[2,7], Yumi Matsuda[1], Keisuke Sato[1], Mai Yokoji[1], Benso Sulijaya[1], Tomoki Maekawa[2], Tatsuo Ushiki[3], Yoshikazu Mikami[3], Manabu Hayatsu [3], Yusuke Mizutani[3], Shigenobu Kishino[4], Jun Ogawa[4], Makoto Arita[5], Koichi Tabeta[6], Takeyasu Maeda[2], Kazuhisa Yamazaki[1,7]

Affiliations

[1] Research Unit for Oral-Systemic Connection, Division of Oral Science for Health Promotion, Niigata University Graduate School of Medical and Dental Sciences, Niigata, Japan.

[2] Research Center for Advanced Oral Science, Niigata University Graduate School of Medical and Dental Sciences, Niigata, Japan.

[3] Division of Microscopic Anatomy and Bio-imaging, Niigata University Graduate School of Medical and Dental Sciences, Niigata, Japan.

[4] Division of Applied Life Sciences, Graduate School of Agriculture, Kyoto University, Kyoto, Japan.

[5] Laboratory for Metabolomics, RIKEN Center for Integrative Medical Sciences, Kanagawa, Japan

[6] Division of Periodontology, Department of Oral Biological Science, Niigata University Faculty of Dentistry, Niigata, Japan.

[7] Corresponding author

Supplementary Figure 1

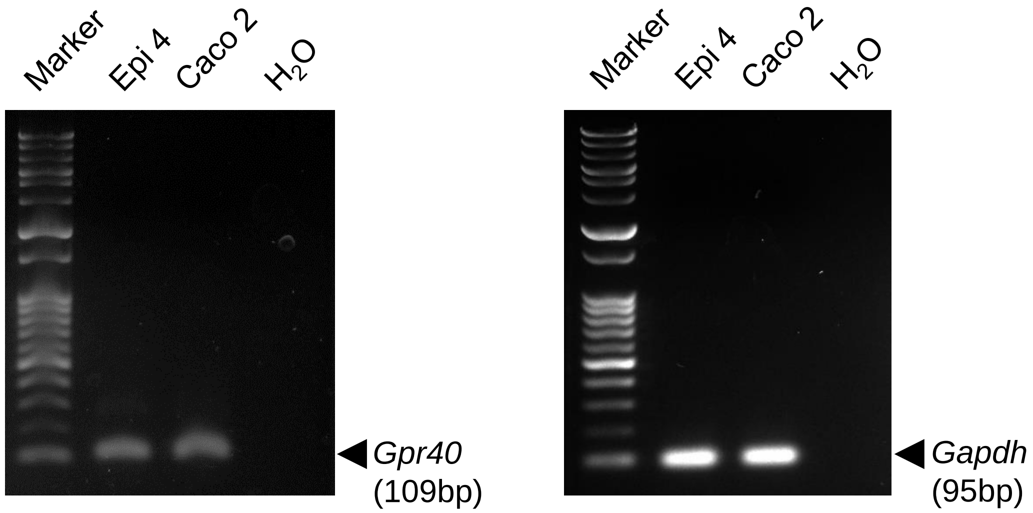

The full-length gel image for Figure1A.
Agarose gel electrophoresis of RT-PCR products for *Gpr40* (left panel) and *Gapdh* (right panel). Caco 2 was used as a positive control. H_2O was used as a negative control.

Supplementary Figure 2

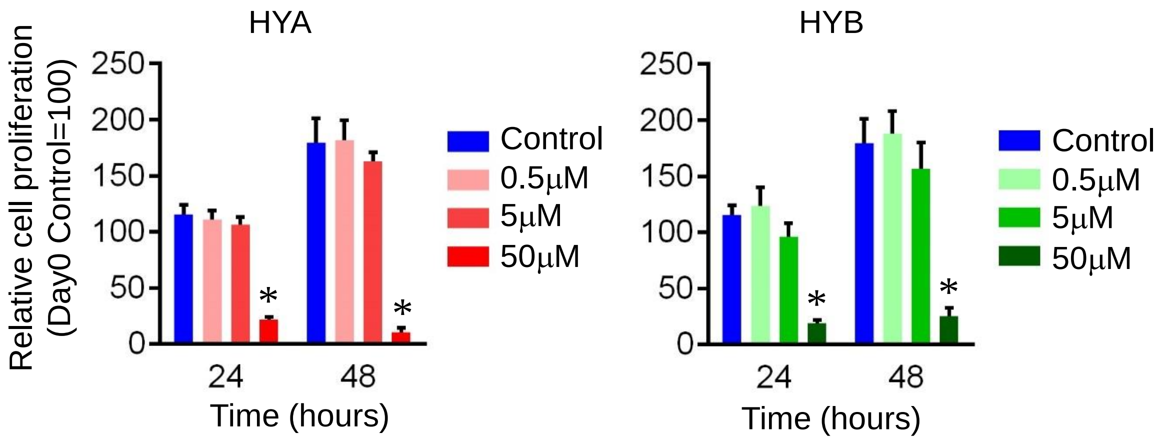

Optimization of concentration of metabolites for *in vitro* study.
In vitro cytotoxic activities of metabolites at the indicated concentration were assessed using MTT assay. The relative cell proliferation of Epi4 cells was compared at the indicated time point. Less than 5μM of HYA/HYB did not show any cytotoxicity at both time points. (n = 4 in each group). All data are mean ± SD. $p < 0.01$ versus control at same time point, by ANOVA.

Supplementary Figure 3

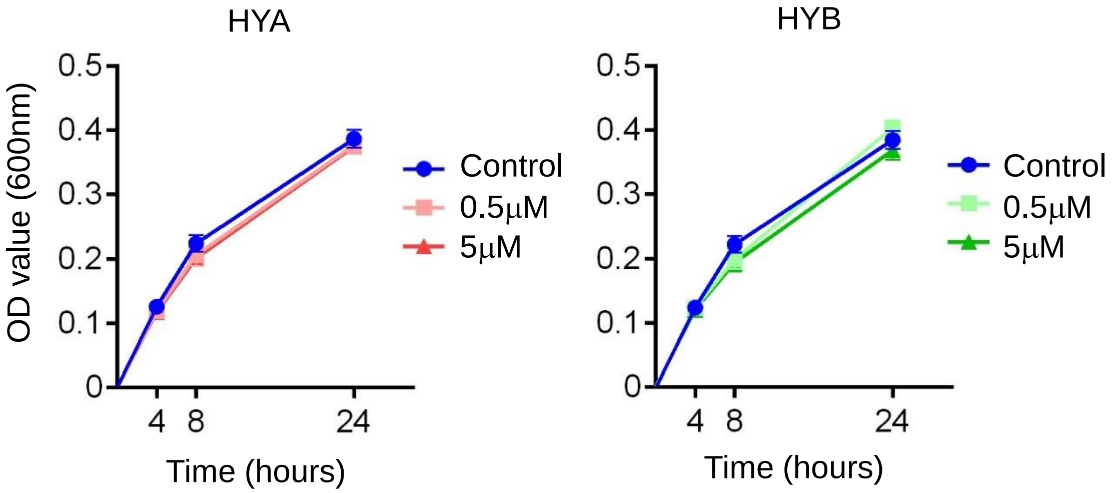

No antimicrobial effect of HYA/HYB against *P. gingivalis*.

P. gingivalis was grown in liquid medium in the presence of the indicated concentration of HYA/HYB. Growth rate and bacterial concentration were determined by measuring OD value at 600 nm at the indicated time points. Neither HYA nor HYB showed any antimicrobial activities against *P. gingivalis* at all time points. (n = 5 in each group). All data are mean ± SD.

Supplementary Figure 4

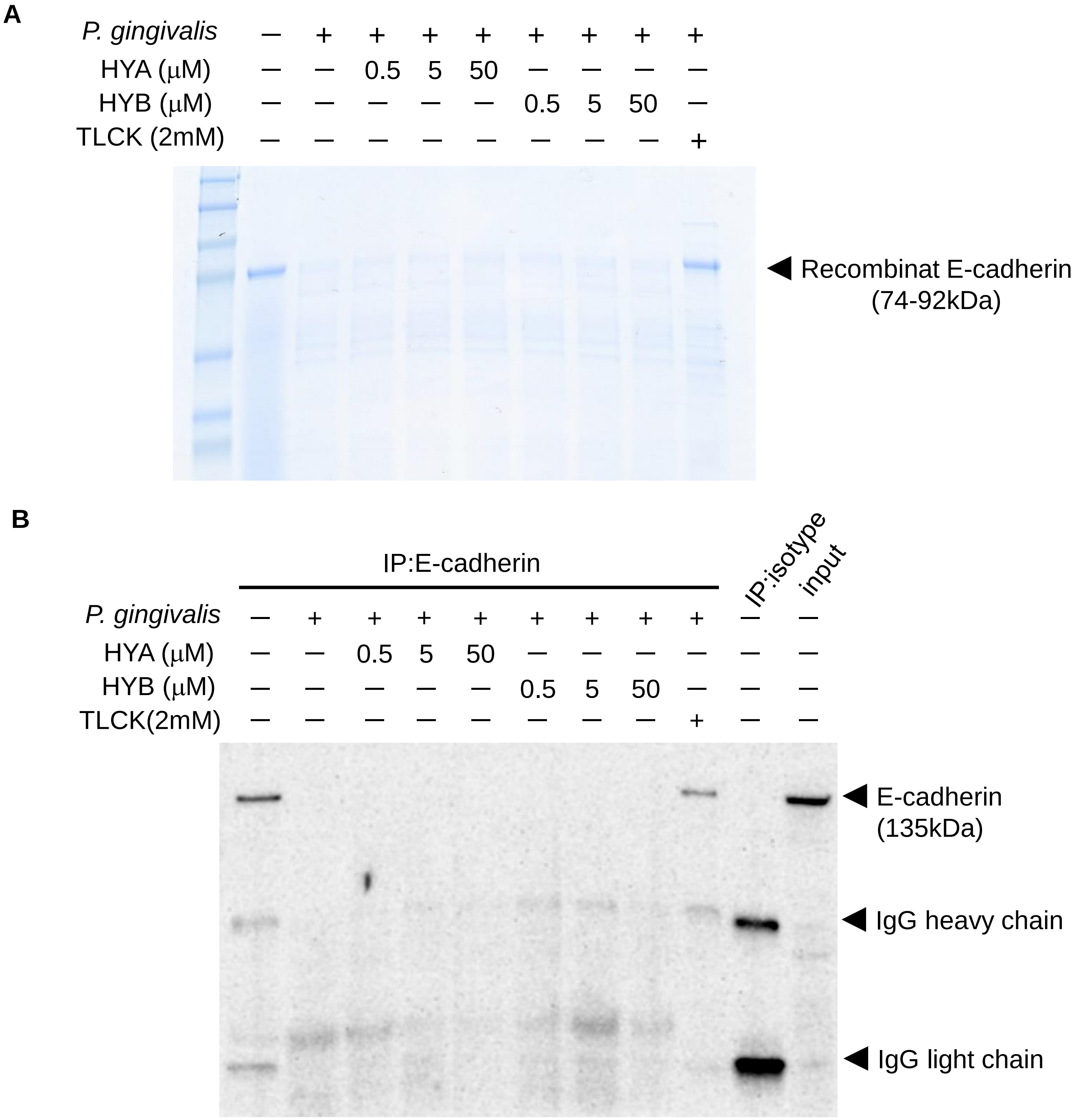

No anti-proteolytic activities of HYA against *P. gingivalis*-derived protease.
The effect of HYA/HYB in the degradation of E-cadherin protein induced by *P. gingivalis*-derived protease *in vitro*. (A) Recombinat human E-cadherin and (B) immunoparticipated E-cadherin of Epi 4 cells were incubated with live-*P. gingivalis* (MOI:100) for 4 hours with or without the preincubation of indicated concentration of HYA/HYB for 30 mins. Loaded proteins were visualized by Coomassie-Blue-staining in upper panel and western blotting in lower panel, respectively. Arrows on the left side indicate the predicted size of E-cadherin. Nα-tosyl-L-lysine chloromethyl ketone hydrochloride (TLCK) served as a positive control of protease inhibitor.

Supplementary Figure 5

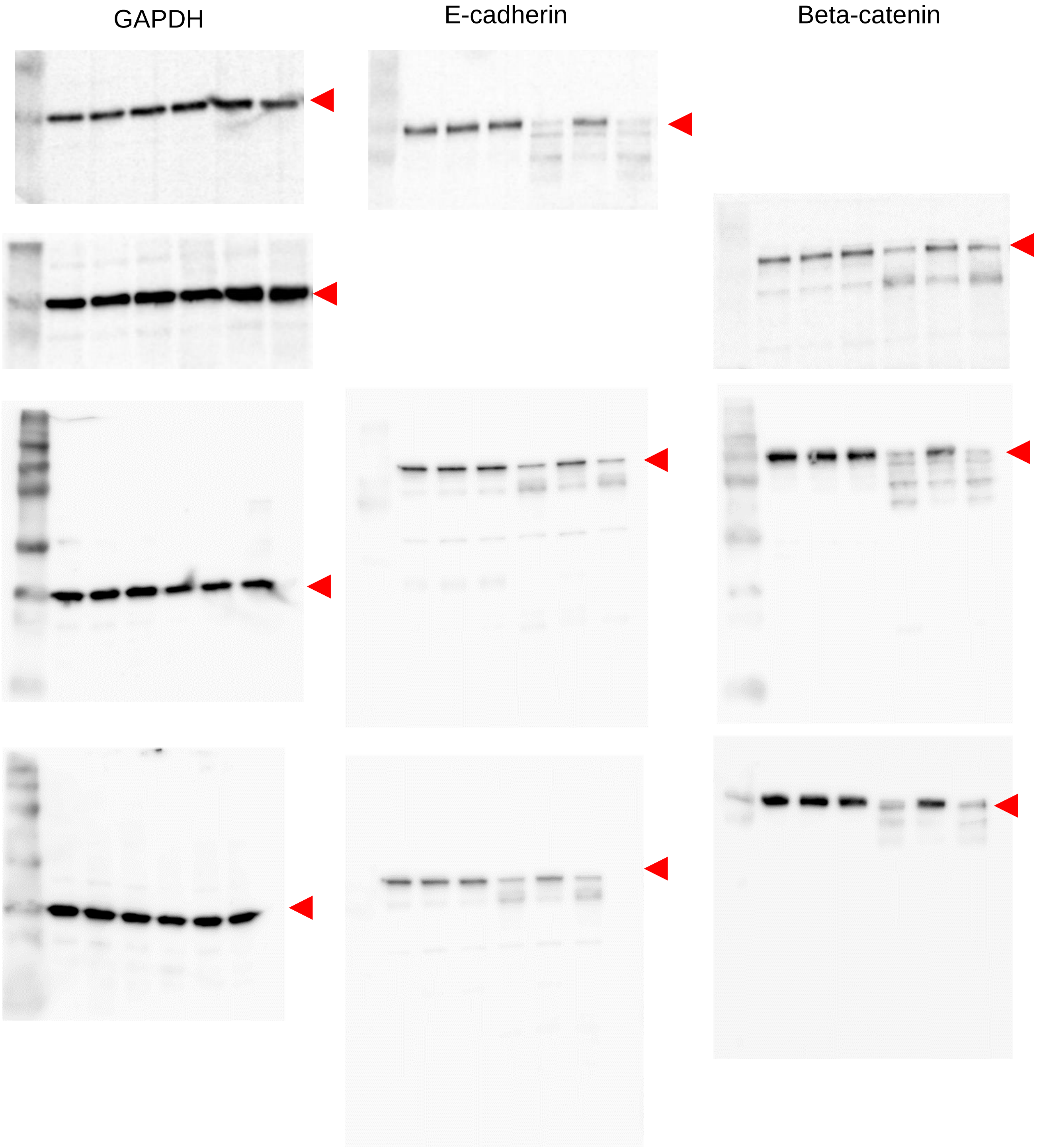

Full-length immunoblot images for Figure 4A.

Supplementary Figure 6

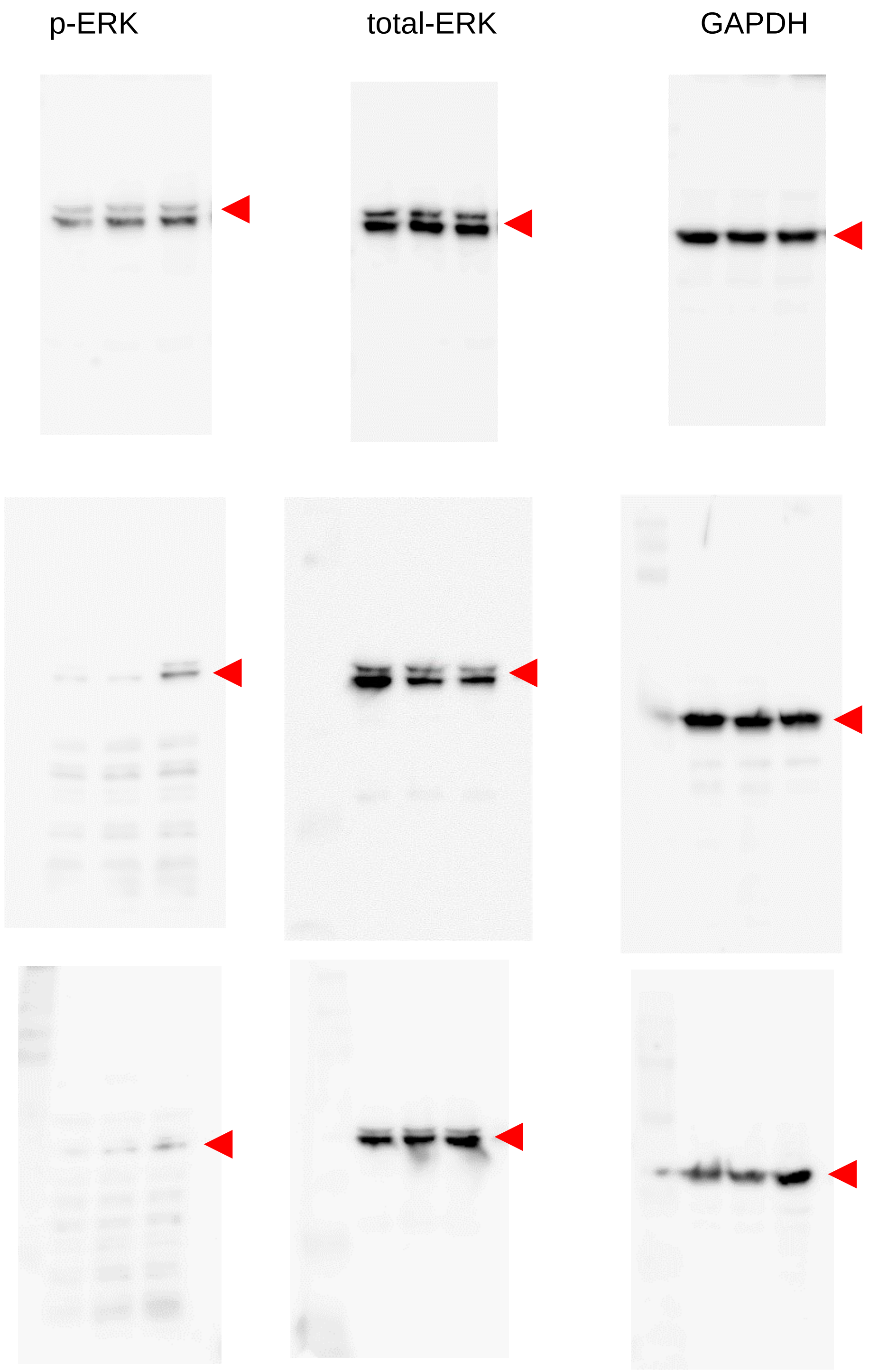

Full-length immunoblot images for Figure 4B.

Supplementary Figure 7

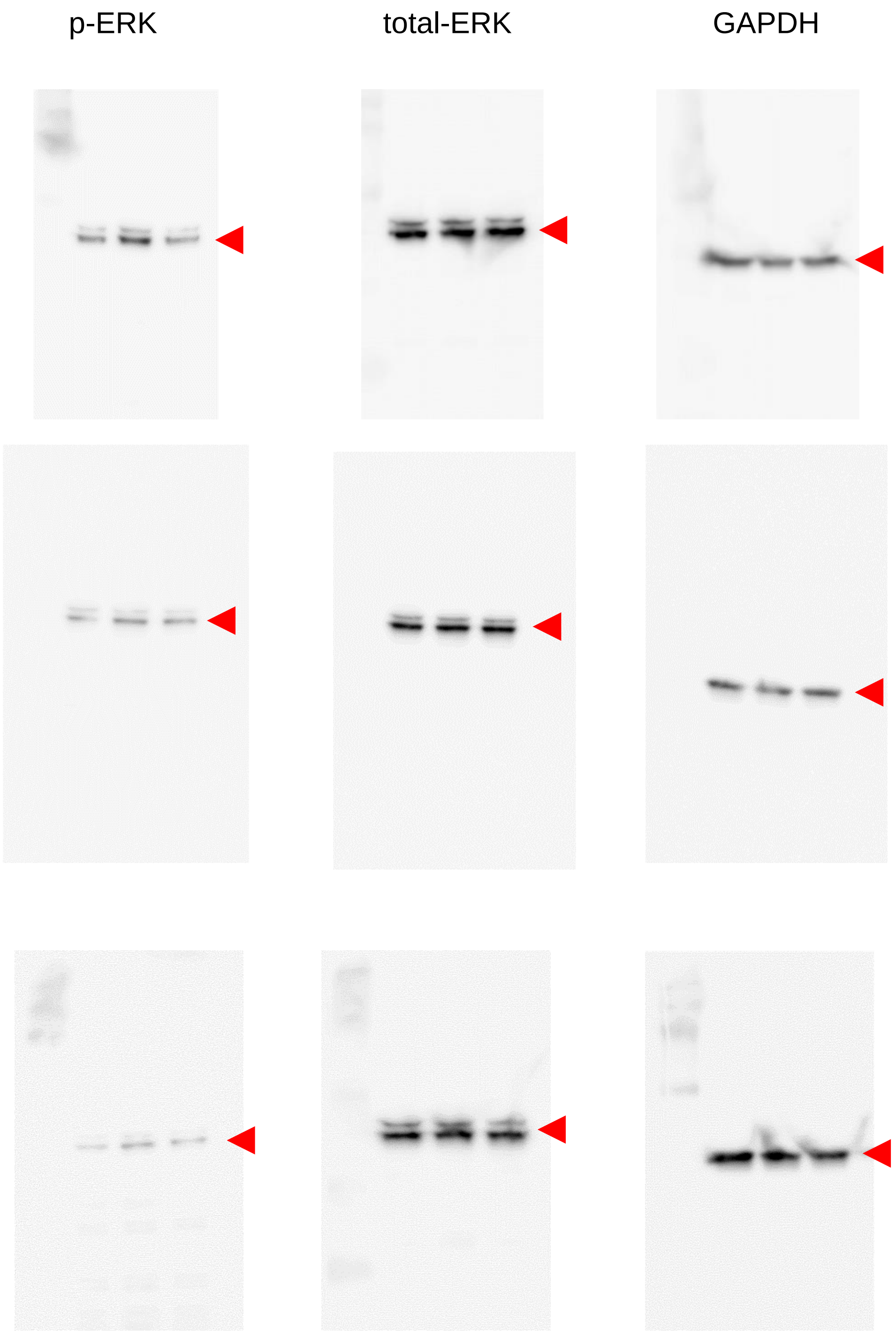

Full-length immunoblot images for Figure 4C.

Supplementary Figure 8

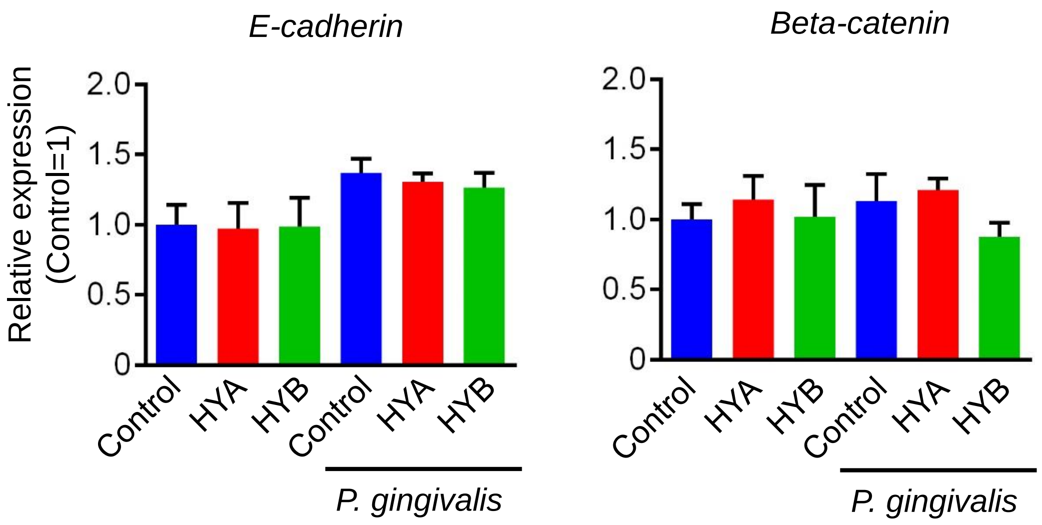

No alteration on E-cadherin/Beta-catenin mRNA level.

Quantification of indicated mRNA expression of Epi 4 cells at 4 h after stimulation, with or without HYA/HYB preincubation (5 µM) for 30 min. (n = 3 in each group). All data are presented as mean ± SD.

Supplementary Figure 9

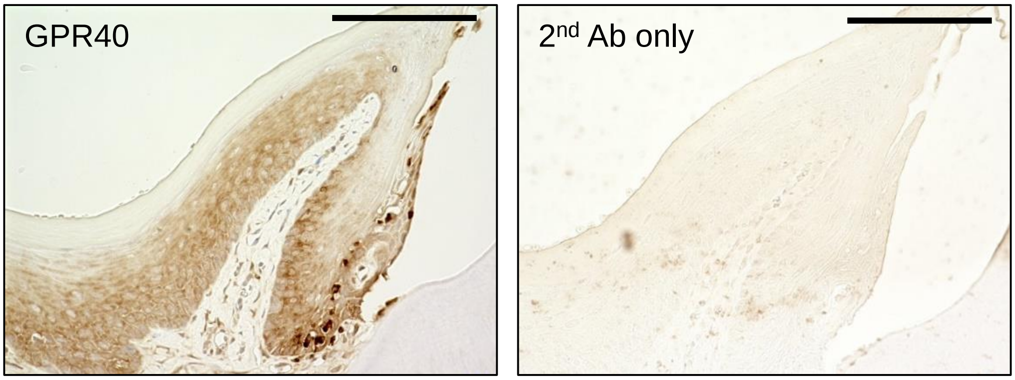

GPR40 expression in the gingival epithelium.
Representative immunohistochemical staining of murine gingival sections with anti-GPR40 antibody. GPR40 immunoreactivity was detected in the basal and suprabasal layers as well in junctional epithelium. The right panels are sections without primary antibody, which served as negative controls Scale bars represent 100 μm.

Supplementary Figure 10

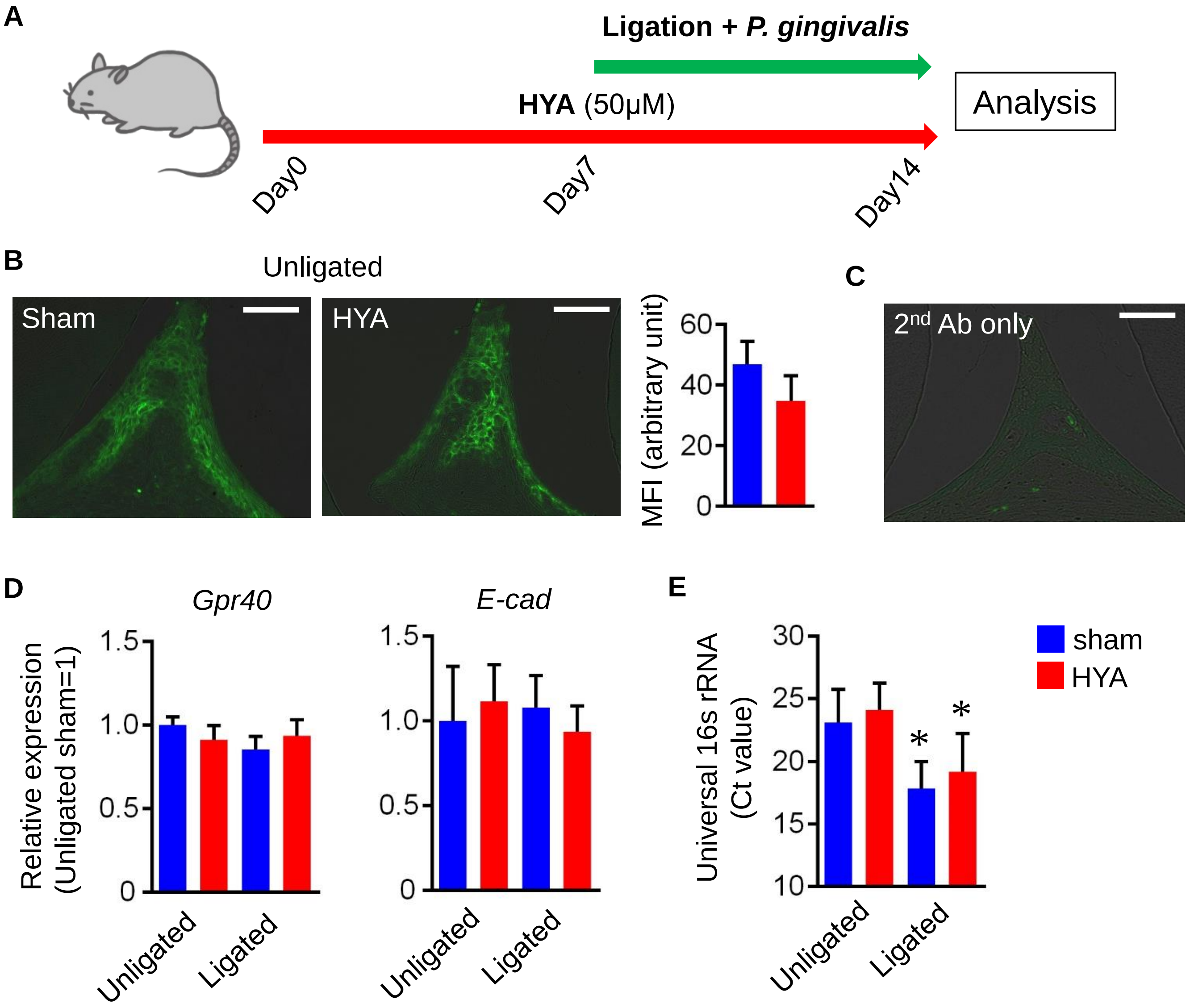

Effect of HYA treatment in the ligature-induced periodontitis model.
(A) Experimental design of HYA treatments in the mice model. (B) Representative immunofluorescence staining of epithelial E-cadherin and quantification. (n=3 in each group) Scale bars represent 100 µm. (C) Validation of E-cadherin antibody for immunofluorescence staining. (D) Quantification of indicated mRNA expression in gingival tissue. (n=6 in each group) (E) Quantification of bacterial abundance was performed by PCR using universal 16s rRNA primerson Day14. Subgingival plaque samples were obtained from each mouse by placing sterile paper points. (n=6 in each group). All data are mean ± SD. *$p < 0.05$ vs unligated sham group, by ANOVA.

Supplementary Figure 11

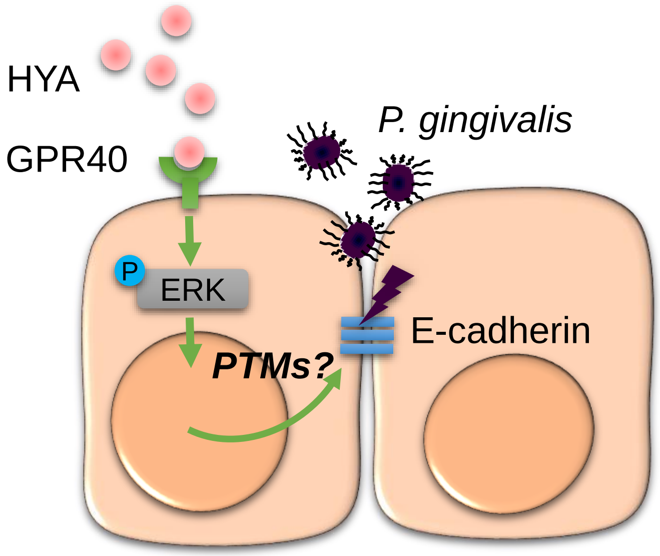

A schema of our proposed mechanism. Post-translational modifications (PTMs) on E-cadherin mediated by HYA-GPR40-ERK signaling suppress the degradation of E-cadherin proteins in Epi 4.
